# RETRACTION: Parallel Declines in Abundance of Insects and Insectivorous Birds in Denmark Over 22 Years

**DOI:** 10.1002/ece3.73864

**Published:** 2026-06-11

## Abstract

**RETRACTION**: MøllerA. P. , “Parallel Declines in Abundance of Insects and Insectivorous Birds in Denmark Over 22 Years,” Ecology and Evolution 9, no. 11 (2019): 6581‐6587, 10.1002/ece3.5236.31236245 PMC6580276

The above article, published online on 15 May 2019 in Wiley Online Library (wileyonlinelibrary.com), has been retracted by agreement between the journal Editors‐in‐Chief, Allen Moore and Arley Muth; and John Wiley & Sons, Ltd. The retraction has been agreed due to concerns raised by third parties. Specifically, multiple inconsistencies within the data have been identified, including duplications affecting large portions of the dataset. The author did not respond to our requests for comments. Accordingly, the article has been retracted as the editors consider its results and conclusions to be invalid. The author has been informed of the retraction decision but was not unavailable for final confirmation.

**RETRACTION**: A. P. Møller , “Parallel Declines in Abundance of Insects and Insectivorous Birds in Denmark Over 22 Years,” Ecology and Evolution 9, no. 11 (2019): 6581‐6587, 10.1002/ece3.5236.31236245 PMC6580276

The above article, published online on 15 May 2019 in Wiley Online Library (wileyonlinelibrary.com), has been retracted by agreement between the journal Editors‐in‐Chief, Allen Moore and Arley Muth; and John Wiley & Sons, Ltd. The retraction has been agreed due to concerns raised by third parties. Specifically, multiple inconsistencies within the data have been identified, including duplications affecting large portions of the dataset. The author did not respond to our requests for comments. Accordingly, the article has been retracted as the editors consider its results and conclusions to be invalid. The author has been informed of the retraction decision but was not unavailable for final confirmation.

